# *TP53* mutation and immunohistochemical p53 expression characteristics in diffuse large B–cell lymphoma

**DOI:** 10.3389/fonc.2025.1550207

**Published:** 2025-04-28

**Authors:** Yiping Jin, Yi Wang, Lu Wang, He Zhang, Beibei Ren, Jiawen Zheng, Qingxin Xia, Yanyan Liu

**Affiliations:** ^1^ Department of Pathology, Affiliated Cancer Hospital of Zhengzhou University & Henan Cancer Hospital, Zhengzhou, Henan, China; ^2^ Department of Molecular pathology, Affiliated Cancer Hospital of Zhengzhou University & Henan Cancer Hospital, Zhengzhou, Henan, China; ^3^ Department of Internal Medicine, Affiliated Cancer Hospital of Zhengzhou University & Henan Cancer Hospital, Zhengzhou, Henan, China

**Keywords:** *TP53* mutation, diffuse large B–cell lymphoma, p53 protein, immunohistochemistry, next–generation sequencing

## Abstract

Diffuse large B-cell lymphoma (DLBCL) with TP53 mutations has specific clinicopathological features and is usually associated with a poor prognosis. TP53 gene mutations typically lead to aberrant expression patterns of the p53 protein. We studied 123 DLBCL patients at Henan Cancer Hospital, 35.8% (44/123) had TP53 mutations. Analysis of mutation sites in 44 cases of DLBCL patients revealed that the mutations primarily occur in the DNA-binding domain (DBD region) of the encoded p53 protein; among all mutation types, there were 8 truncation or frameshift mutations, and 36 missense mutations. Further, immunohistochemistry (IHC) detected expression levels of p53 protein in 123 DLBCL samples. The mutation results were used as a reference, and receiver operating characteristic (ROC) curve analysis was employed. Ultimately, the expression ratio of 65% and the moderate–strong expression intensity were regarded as the cut–off value, namely high p53 expression or p53 negative (<1%) indicated mutant–type p53 protein. the complete remission (CR) rate of the mutant–type p53 protein group after receiving R–CHOP regimen was 50% (14/28), and the objective response rate (ORR) was 75%, which differed significantly (P<0.01) compared with wild–type p53 protein group [CR rate of 75.86% (66/87) and ORR rate of 89.66%]. Common gene mutations in the mutant–type p53 protein group primarily involve alterations in pathways related to epigenetics, B cell antigen receptor signaling, cell cycle, among others. IHC analysis of the p53 protein is a simple and low-cost approach that can be employed to predict TP53 mutation status and therapy response.

## Introduction

*TP53* is an important tumor suppressor gene on the short arm of chromosome 17 (17p13.1). The encoded p53 protein is involved in apoptosis, DNA repair, cell metabolism, differentiation, and development (1). Somatic mutations in the *TP53* gene are among the most common alterations in human cancers, occurring in nearly all tumor types (2). Compared with solid tumors, the *TP53* mutation frequency in hematologic tumors is relatively low (5–15%); however, its mutations cover all hematologic tumor types. The mutation spectrum and frequency of *TP53* vary according to different tumor types and have been consistently associated with poor prognosis (3).

Diffuse large B-cell lymphoma (DLBCL) stands as the most prevalent type of non-Hodgkin lymphoma (4), characterized by significant biological heterogeneity. Current evidence indicates that following the standard first–line treatment regimen involving cyclophosphamide, vincristine, rituximab, doxorubicin, and prednisone (R-CHOP) for DLBCL patients, about 60% can obtain long–term survival (5), however about 40% still have primary drug resistance or recurrence. Therefore, early risk stratification in individuals with DLBCL is essential for prognostic evaluation and personalized treatment.

With advancements in genome and transcriptome detection and analysis techniques, the molecular classification of DLBCL has also progressively enhanced. From the cell of origin (COO) classification (6) to the now five and seven classifications among others (7–10), different types such as MCD type (based on the co-occurrence of MYD88L265P and CD79B mutations), BN2 type (based on BCL6 fusions and NOTCH2 mutations), EZB type (based on EZH2 mutations and BCL2 translocations), N1 type (based on NOTCH1 mutations), and A53 type (aneuploid with TP53 inactivation), all have their characteristic gene alterations. The key gene alteration of the A53 type is *TP53* mutation. Studies have confirmed that *TP53* somatic mutation is present in approximately 20% of DLBCL (11, 12), and *TP53* gene mutation is typically linked with poor prognosis (13).

In this research, next–generation sequencing (NGS) was conducted on paraffin samples of individuals with DLBCL who had undergone R–CHOP or R–CHOP–like treatments, and characteristic analysis was conducted in terms of *TP53* gene alterations to compare with clinical characteristics. Furthermore, the immunohistochemistry (IHC) technique was employed for p53 protein detection and its comparison with *TP53* mutation status. This indicates that IHC analysis of the p53 protein, serving as a substitute for *TP53* mutation status, is crucial for the prognostic stratification of patients.

## Materials and methods

### Patients and samples

This research involved 123 individuals with newly diagnosed DLBCL at Henan Cancer Hospital from March 2018 to March 2022. The diagnosis should be confirmed by at least two pathologists following the criteria outlined in the World Health Organization classification (14). Clinical characteristics of all patients were collected, including sex, age, site of disease, international prognostic index (IPI) score, ECOG score, LDH elevation, number of extranodal involvement, B symptoms, Ann Arbor stage, treatment regimens, and long–term survival data. Hans’ algorithm was utilized to categorize DLBCL cases into GC and non–GC phenotypes (15). Most patients had received at least 4 cycles of R-CHOP or R-CHOP-like treatment regimens. Efficacy evaluation included complete response (CR), partial response (PR), stable disease (SD), and progressive disease (PD); primary endpoints included complete remission rate (CRR), progression–free survival (PFS), and overall survival (OS). The approval for this research was provided by the Ethics Review Committee of our hospital as per the Declaration of Helsinki.

### Immunohistochemistry

An indirect immune-peroxidase method was employed, utilizing antibodies against CD10 (Abcam, 1:500), BCL6 (Abcam, 1:500), MUM1 (Abcam 1:250), BCL2 (Abcam, 1:250), MYC (Abcam, 1:250). The cut-off for c-MYC positivity was defined as more than 40% of tumor cells exhibiting immunoreactivity, while thresholds for BCL2 were set at more than 50%, following previously established criteria (16). When both BCL2 and c-MYC are positive, it is considered double expression. Unstained formalin-fixed paraffin-embedded (FFPE) sections obtained from tumor specimens collected at the time of diagnosis underwent IHC staining using p53 antibodies of MX008 (mouse monoclonal antibody; MXB^®^ Biotechnologies).

The evaluation of p53 IHC staining patterns was conducted by central review of p53 stained whole slide sections. And only the tumor cells with clear staining are counted as a proportion of all tumor cells. P53 stain intensity was classified as weak, moderate, or intense. Two pathologists, blinded to the clinical data, independently evaluated all samples. Any discrepancies were resolved through a collaborative review utilizing a multi-head microscope.

### Next–generation sequencing

Genomic DNA was extracted from formalin-fixed paraffin embedded tumor tissues of DLBCL patients using the QIAamp DNA FFPE Tissue kit (Qiagen). 400 ng of genomic DNA was fragmented using a Bioruptor Diagenode Plus sonicator. End-repair, A-tailing, adapter ligation, PCR reactions and target enrichment were performed then following the manufacturer’s recommended protocols. DNA libraries were hybridized with a commercial gene panel, which includes 93 lymphoma-related genes including *TP53* and covers most of the exons and intron boundaries within at least ±20 bases of genes in the panel list. Final libraries were quantified using a Qubit High Sensitivity kit (Thermo Fisher Scientific) with the quality of the library being assessed using a Bioanalyzer High Sensitivity DNA chip (Agilent Technologies). DNA libraries were sequenced in the department of Molecular Pathology of Henan Cancer Hospital using a 100bp paired-end configuration on a MGISEQ-2000 (MGI Tech, Shenzhen, China) sequencer, with an average depth of at least 500-fold coverage. Sequencing data was aligned to the human reference genome (build hg37) after removal of low-quality reads, using the bwa-mem tool (v0.7.15) with default parameters (17). VarDict (v1.4.6) (18) and Varscan (v2.4.2) (19) were utilized to call SNVs (Single Nucleotide Variants) and small InDels (Insertions and Deletions of length less than 20 base pairs) from the BAM files. The following criteria were applied to filter variants which were identified by both the callers above: the aligned reads depth of the variants should be over 500 and the variant frequency be over 2%. The resulting variants were annotated by SnpEff (v4.3) (20) and then integrated into a unified database framework using Gemini (v0.19.1) (21). The population allele frequency of the final mutations in the 1000G, ESP or ExAC database was required to be lower than 5% for exclusion of potential germline mutations.

### Statistical analysis

Statistical analysis was carried out using Statistical Package for the Social Sciences (SPSS) 24.0 software (IBM, SPSS Inc., Chicago, USA). Statistically significant differences were evaluated utilizing the chi–squared test. Correlations were analyzed using Spearman’s rank correlation coefficient. Receiver operating characteristic (ROC) curve analysis was performed to assess the discriminatory accuracy of p53 protein overexpression in predicting *TP53* mutation status. Estimates of PFS and OS were calculated according to the Kaplan–Meier method and compared between groups using the log–rank test. The results were considered statistically significant when *P* values were *< 0.05*.

## Results

### Clinical characteristics

Of the 123 patients analyzed, 53.7% (66/123) were males. The median age recorded was 57 years (range: 12–83 years), with 43.1% (53/123) falling into the age group over 60 years. Among other clinical characteristics observed, (56.1%, 69/123) were classified as stage III–IV, while (40.6%, 50/123) exhibited higher IPI scores. Utilizing the Hans algorithm for classification revealed that non–germinal center subtypes constituted 52.0% (64/123) of the cases. MYC protein positivity was observed in 36.6% (45/123) of cases, while double expressions were present in 31.7% (39/123), and *TP53* mutation was identified in 35.8% (44/123) of cases (**Table 1**).

**Table 1:** Cohort description-clinical and pathological characteristics.

Median age at diagnosis	57 (12-83)
>60	43.1% (53/123)
≤60	56.9% (70/123)
Gender	
Male	53.7% (66/123)
Female	46.3 (57/123)
Ann Arbor staging classification	
I-II	43.9% (54/123)
III-IV	56.1% (69/123)
R-IPI class	
0	16.3% (20/123)
1-2	43.1 % (53/123)
3-5	40.6% (50/123)
ECOG high status (>1 point)	20.3% (25/123)
LDH elevation	41.5% (51/123)
Extranodal involvement (>1)	39.8% (49/123)
Presence of B symptoms	15.4% (19/123)
DLBCL subtype (Hans’ algorithm)	
GCB	48.0% (59/123)
Non-GCB	52.0% (64/123)
c-MYC	
Positive	36.6% (45/123)
Negative	63.4% (78/123)
DEL	31.7% (39/123)
TP53 mutation	35.8% (44/123)

R-IPI, revised international prognostic index; ECOG, Eastern Cooperative Oncology Group; GCB, germinal center B–Cell–like subtype; Non-GCB, non-germinal center B–Cell–like subtype; DEL, double expressions lymphoma.

### Characteristics of *TP53* mutation

**Figure 1:**
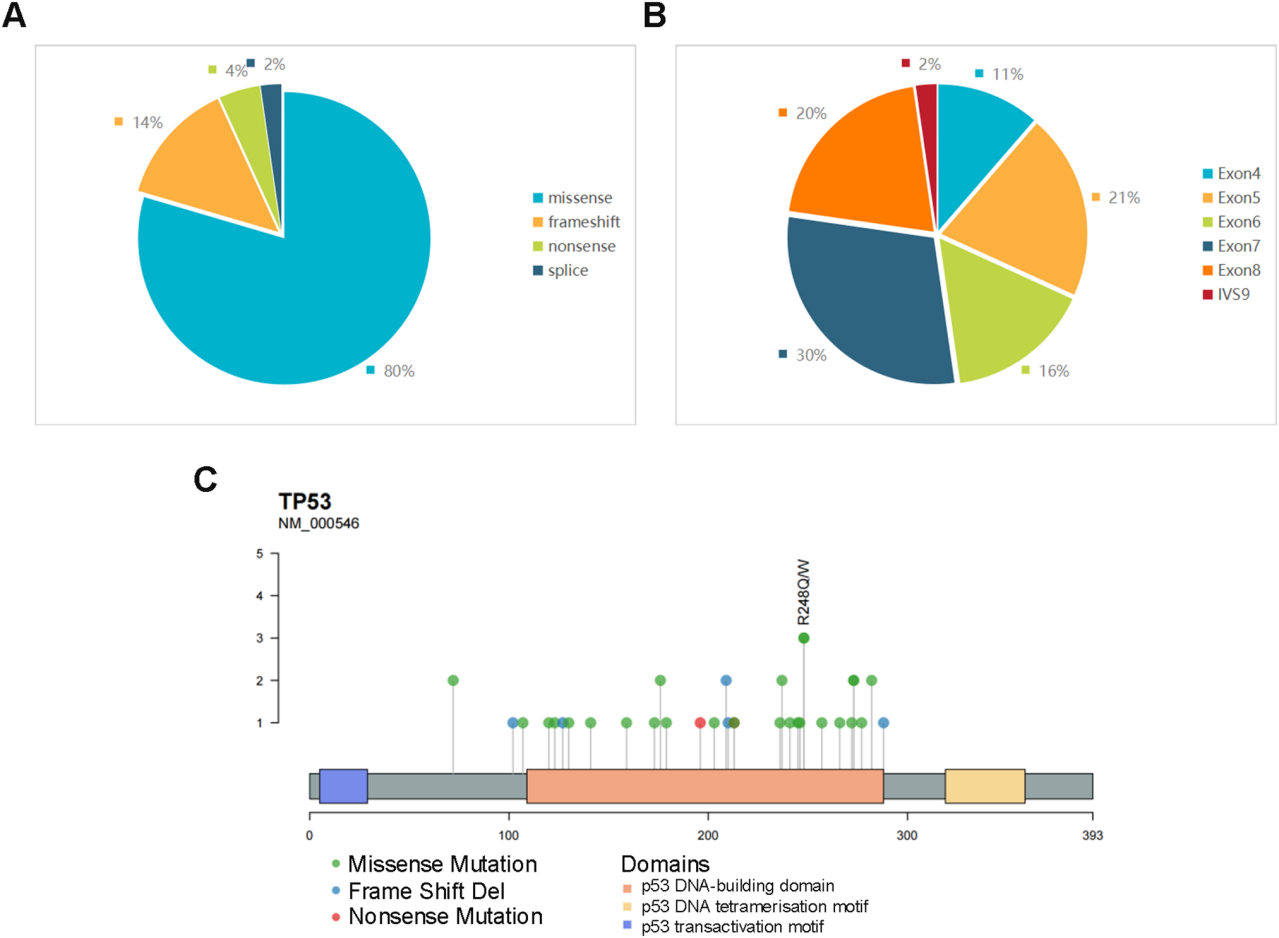
**(A)** Proportions of different mutation types of TP53. **(B)** Distribution proportion of *TP53* mutations in different exons and introns. **(C)** Codon lollipop chart of *TP53* mutations, marking the site with the highest mutation frequency in the cohort.

NGS was performed on 123 patients with DLBCL, and *TP53* mutation was observed in 44 patients. The most common mutation type was missense mutations (n=35, 80%). In addition, 6 presented frameshift mutation, 2 nonsense mutations, and 1 splicing mutation (**Figure 1**). Mutations were predominantly observed within the exon region, representing 98% of the occurrences, with distribution spanning from exons 4 to 8. Notably, the most frequent location for these mutations was found to be exon 7 (**Figure 1**). The distribution of these exons and codons is shown in **Figure 1**. It was observed that most mutations were in p53 DNA–binding domains, comprising a total of 39 mutations (88.6%). Notably, codon 248 exhibited the highest frequency of mutations, with other commonly affected codons, including 72, 176, 209, 237, 273, and 282 (**Supplementary Table 1**). Certain mutations, such as those at codons 273 and 282, were identified as hot spots for *TP53* mutations, prevalent across a wide spectrum of human cancers (22).

### Correlation between IHC p53 expression and *TP53* mutation

**Figure 2:**
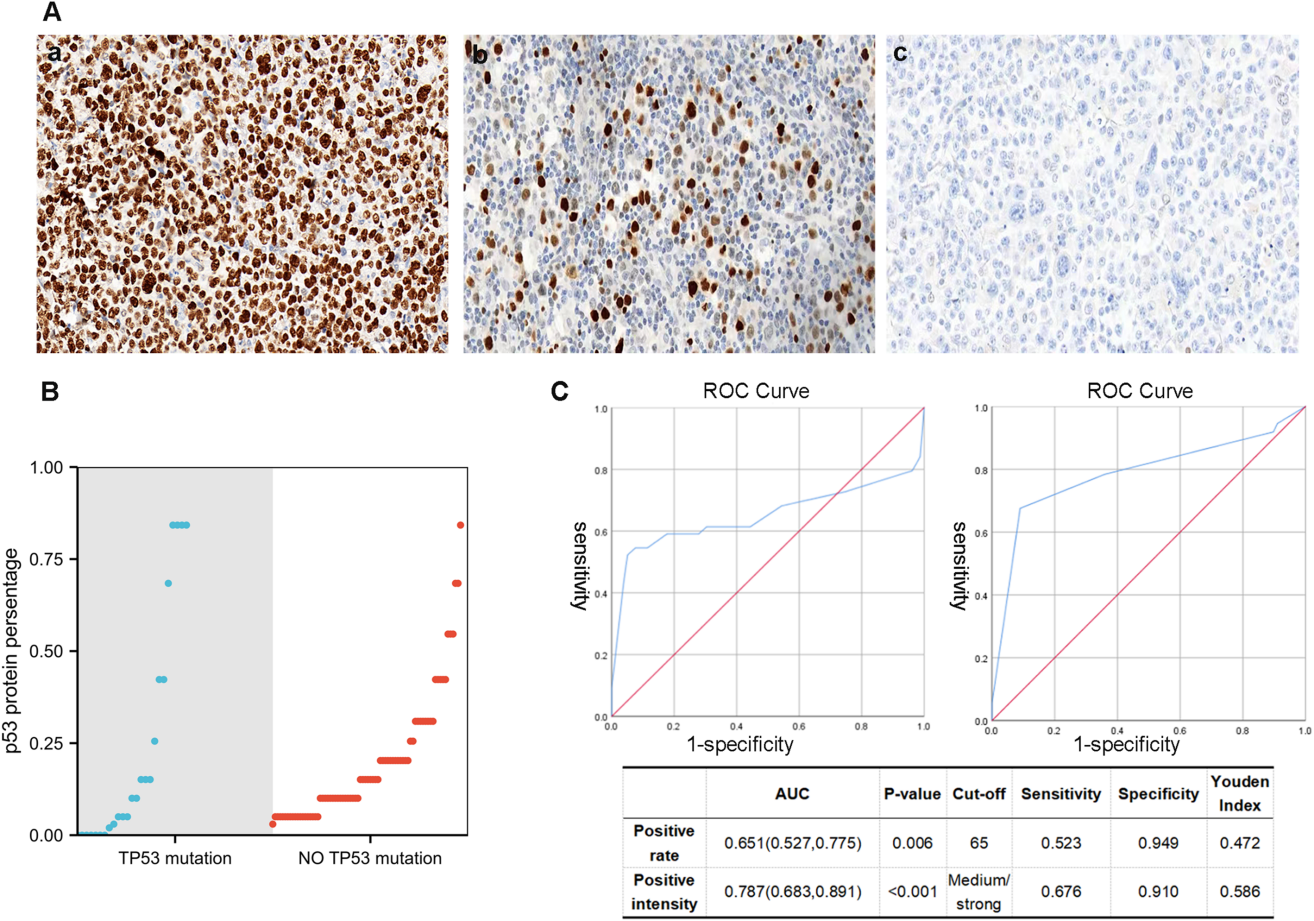
**(A)** Different staining states of p53 (IHC), (a) 90% moderate–strong staining (×40), (b) 20% weak–moderate–strong staining (×40), (b) <1% staining. **(B)** The relationship between *TP53* mutation and p53 IHC percentage. **(C)** For the cut–off value of the high–expression group, ROC curve analysis is employed. When the p53 positive rate is 65%, and the positive intensity is moderate and/or strong, it would have the largest Youden’s index.

The IHC method, being simple and cost-effective, would greatly facilitate our work if it proves to be a suitable alternative to molecular assays. Therefore, IHC was employed to detect p53 protein expression levels in 123 DLBCL samples. According to their positive intensity and rate, patients were assigned to the negative group (<1%), low–expression group, and high–expression group (**Figure 2**), using the *TP53* mutation results in NGS as a reference (**Figure 2**; **Supplementary Table 2**). In the negative group, there were 4 cases, of which 3 were frameshift mutation and 1 was nonsense mutation. The high–expression group primarily corresponded to the missense mutation of the *TP53* gene (**Supplementary Table 1**). For the cut–off value of the high–expression group, ROC curve analysis was performed. When the p53 positive rate was 65%, and the positive intensity was moderate and/or strong, it exhibited the largest Youden’s index (**Figure 2**). Therefore, in this study, a cut-off value of 65% expression ratio combined with moderate to strong expression intensity was employed to define high p53 expression. If high expression or complete absence of p53 is indicated through immunohistochemical methods, suggesting the presence of mutant-type p53 protein, we designate it as MUT-p53 (mutant–type p53). Conversely, low expression suggests the presence of wild-type p53 protein, which we term WT-p53 (wild-type p53). The sensitivity of the p53 protein mutation type to *TP53* gene mutation cases was 61.4%, the specificity was 93.7%, and the accuracy was 82.1%. The corresponding positive and negative predictive values were 84.4% and 82.3% respectively (**Supplementary Table 2**).

### Mutant–type p53 (IHC) and wild-type p53 (IHC)

IHC was employed to allocate 123 patients with DLBCL to two groups: mutant–type p53 (MUT-p53) and wild–type p53 (WT-p53) groups, of which MUT–p53 accounted for 26.0% (32/123) and WT–p53 accounted for 74.0% (91/123). There were no significant differences in age, gender, IPI score, Han’s classification, and double expression between the two groups. The MUT–p53 group was primarily in the early stage of the disease (I–II) at initial treatment, whereas the WT–p53 group was primarily in the middle and advanced stages (III–IV). Hence, a significant difference was observed (*P=0.014*). The MUT-p53 group of patients is more likely to experience B symptoms (*P*=0.021) (**Table 2**).

**Table 2:** Clinical differences between mutant-type p53 protein and wild-type groups.

Parameters		WT-p53 (IHC)	MUT-p53 (IHC)	*P*
Median age at diagnosis	>60	42	11	0.250
	≤60	49	21	
Gender	Male	49	17	0.944
	Female	42	15	
Ann Arbor staging classification	I-II	34	20	0.014*
	III-IV	57	12	
R-IPI class	0	11	9	0.093
	1-2	40	13	
	3-5	40	10	
ECOG status	>1	22	3	0.125
	≤1	69	29	
LDH elevation	Yes	36	15	0.430
	No	55	17	
Extranodal involvement	>1	40	9	0.116
	≤1	51	23	
B symptoms	presence	10	9	0.021*
	absence	81	23	
DLBCL subtype (Hans’ algorithm)	GCB	47	12	0.168
	Non-GCB	44	20	
c-MYC	Positive	29	16	0.067
	Negative	62	16	
DEL	Yes	25	14	0.089
	No	66	18	
efficacy evaluation	CR rate	75.86% (66/87)	50% (14/28)	<0.01*
	Non-CR rate	24.16% (21/87)	50% (14/28)	

*Statistically significant at P < 0.05.

R-IPI, revised international prognostic index; ECOG, Eastern Cooperative Oncology Group; GCB, germinal center B–Cell–like subtype; Non-GCB, non-germinal center B–Cell–like subtype; DEL, double expressions lymphoma.

**Figure 3:**
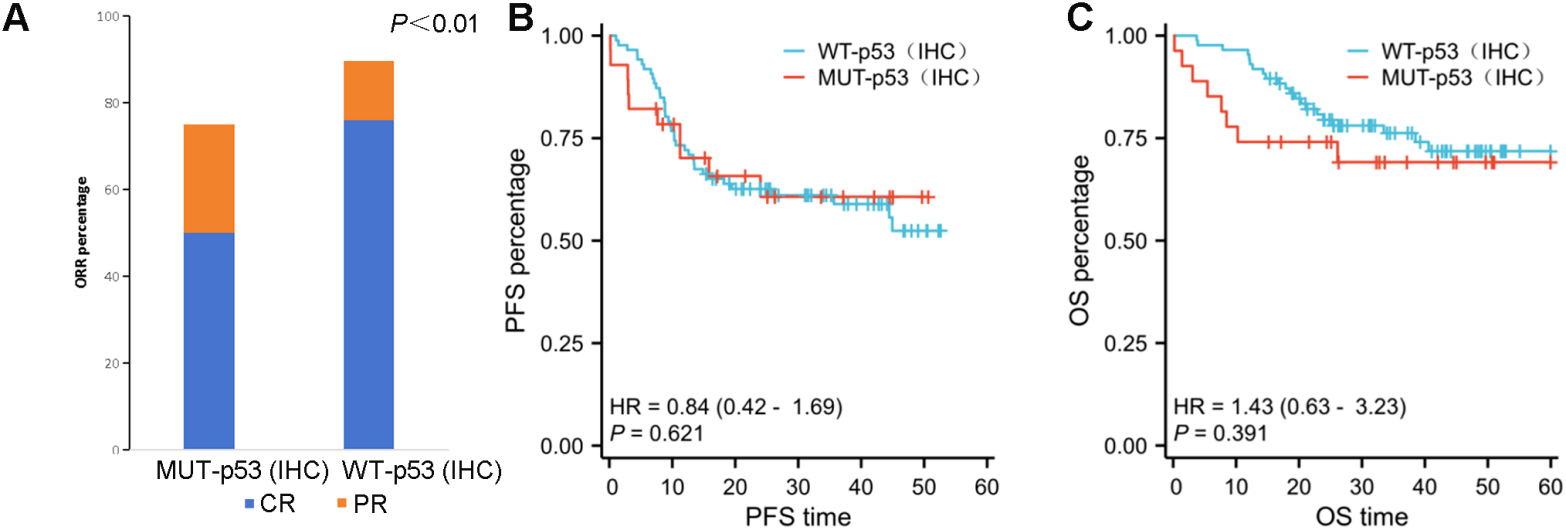
**(A)** The ORR in the WT–p53 (IHC) group is significantly better than that in the MUT–p53 (IHC) group (P ≤ 0.01). **(B, C)** There is no significant difference in PFS **(B)** and OS **(C)** between the MUT–p53 (IHC) group and the WT–p53 (IHC) group.

As compared with the MUT–p53 group, the WT–p53 group was more likely to obtain complete remission. The CRR was 50% (14/28) in the MUT–p53 group and 75.86% (66/87) in the WT–p53 group after receiving the R–CHOP regimen, demonstrating significant differences (*P<0.01*) (**Table 2**). The ORR was 75% in the MUT–p53 group and 89.66% in the WT–p53 group, and the MUT–p53 group was significantly lower than the WT–p53 group (*P<0.01*) (**Figure 3**). The median OS and median PFS of the MUT–p53 group were 26.22 months and 19.41 months, both lower than those of the WT–p53 group (median OS: 32.12 months and median PFS: 24.82 months). However, the survival curve suggested that both groups did not differ significantly in terms of OS (HR 1.43, *P=0.391*) and PFS (HR 0.84, *P=0.621*) (**Figures 3A, B**). *TP53* mutation by NGS also had no significant effect on OS (HR 0.60, *P=0.171*) and PFS (HR 0.70, *P=0.293*) (**Supplementary Figures 1A, B**). In the previous results, we found that the mutant type of p53 protein was more likely to appear in the early stages of the disease. Therefore, we divided the cases into an early-stage disease group and a late-stage disease group for survival analysis. We found that there was no significant difference in OS and PFS between the two groups (**Supplementary Figures 1C–F**).

**Figure 4:**
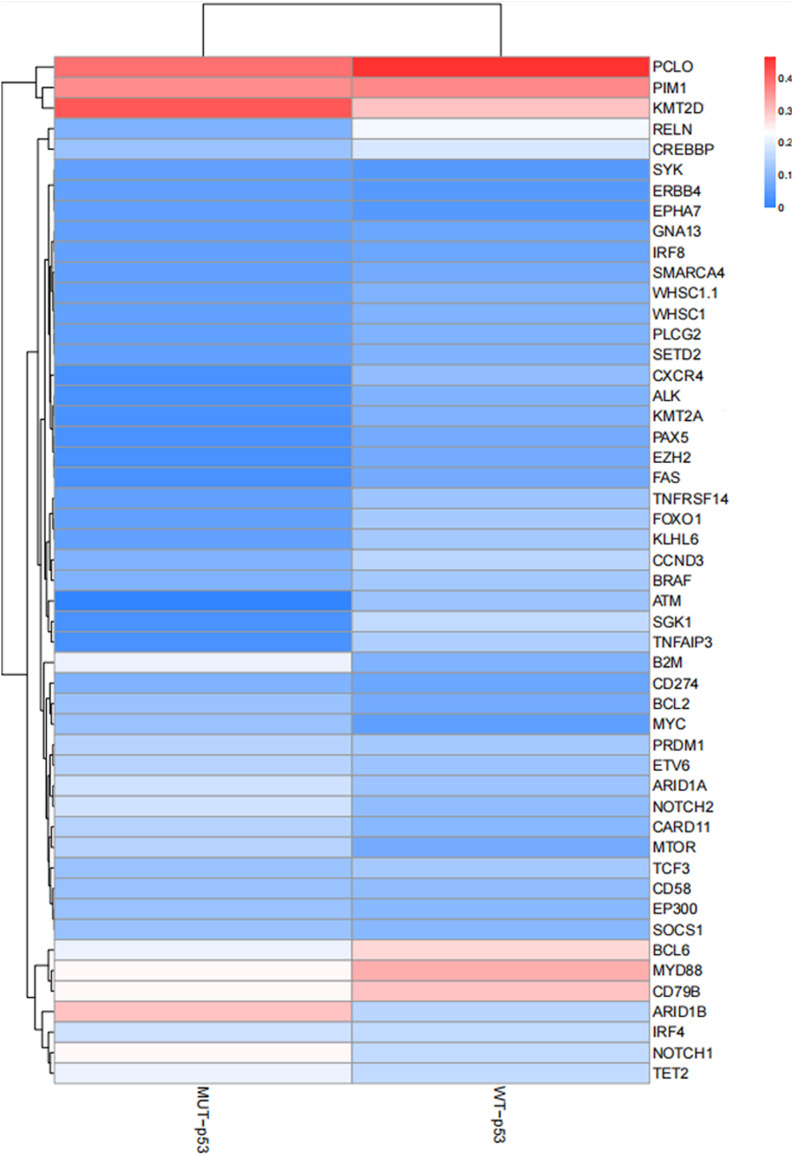
Common gene mutations in the MUT–p53 (IHC) group and the WT–p53 (IHC) group.

High–throughput genetic testing (NGS) was employed to detect a panel of 93 genes in all 123 patients with DLBCL. Common gene mutations in the MUT–p53 (IHC) group included KMT2D, PCLO, PIM1, ARID1B, CD79b, MYD88, and NOTCH1. Conversely, common gene mutations in the WT–p53 (IHC) group included PCLO, PIM1, MYD88, KMT2D, CD79b, BCL6, etc. (**Figure 4**). There was no significant difference in the common genes between the two groups, and mutations primarily involved alterations in different pathways such as epigenetics, cytoskeletal proteins, B cell antigen receptor signaling pathway, and cell cycle.

## Discussion

As the most common type, DLBCL accounts for approximately 30%–40% of all non–Hodgkin lymphomas (23). DLBCL is highly heterogeneous and is primarily characterized by diffuse structure, large cell morphology, and mature B cell phenotype, accompanied by multiple subtypes and genetic characteristics. In recent years, multiple research teams have made progress in examining molecular typing and prognosis of DLBCL. The findings have suggested that during the evolution of cell cloning, genetic mutations continue to accumulate, resulting in gene expression profiles and genomic variation profiles that specifically express tumor biology. Hence, these tumors can be classified according to the genetic characteristics of DLBCL to reveal different risk stratifications and guide clinical precision treatment, which has been a subject of interest in the diagnosis and management of lymphoma.

In 2000 and 2002, Alizadeh et al. (6) and Rosenwald et al. (24) reported that DLBCL was classified as germinal center B cell (GCB)–like and activated B–cell (ABC)–like subtypes based on their gene expression profile characteristics, as well as some unclassified types, which is a COO classification based on gene expression profiles, and has profound significance in the history of DLBCL classification. As research progressed, it became evident that the conventional COO classification still has limitations in evaluating prognosis and treatment response sensitivity in DLBCL. Hence, genetic typing that integrates genetic aberrations such as gene mutations, translocations, and copy number alterations has emerged. Among them, the five–classification method proposed by Shipp et al. and the seven–classification method proposed by Staudt et al. (7, 8, 10) are generally recognized. The different subtypes shown by these two methods overlap in genetic alterations. The C2 type in the five–classification method is consistent with the A53 type (aneuploid with TP53 inactivation) in the seven–classification method, both of which are characterized by *TP53* mutation/deletion. Furthermore, existing evidence suggests that patients with DLBCL having *TP53* mutations receiving rituximab plus CHOP (R–CHOP) exhibit worse OS and PFS compared to those without mutations (13, 25). Identifying DLBCL patients with *TP53* mutations is crucial due to their tendency to experience rapid disease progression, strong chemotherapy resistance, and high recurrence rates.

*TP53* gene is the earliest discovered and most important tumor suppressor gene in humans. The p53 protein it encodes is involved in physiological processes such as cell growth, differentiation, induction of cell cycle arrest, induction of apoptosis, and DNA damage repair (26). The *TP53* gene consists of 11 exons, which encode two transactivation domains, one proline-rich domain, a DNA-binding domain, a hinge region, an oligomerization domain, and a C-terminal domain from the N-terminus to the C-terminus in sequence. The DNA-binding domain corresponds to the amino acid positions 98-298, which is the primary site for mutations (27). *TP53* mutations are prevalent in over half of solid tumors, although the mutation rate in lymphomas tends to be lower. However, in this study, the observed incidence was 35.8%, slightly higher than that reported in other studies. Missense mutations were predominantly observed, with the majority occurring within the p53 DNA–binding domains. Notably, codons 248, 176, 209, 237, 273, and 282 exhibited higher mutation frequencies. These characteristics are consistent with previous findings (13, 28). Although TP53 gene mutations are associated with primary drug resistance, disease recurrence and progression, and poor prognosis, not all TP53 mutation sites possess clear pathogenicity. Some changes in certain sites do not affect the function of the protein; for example, P72R is a clearly identified polymorphic site (29).

NGS for detecting *TP53* gene alterations demands specific laboratory setups, rendering it time-consuming and costly, thus limiting its widespread availability. Consequently, there arises a need for a simpler and more cost-effective alternative to NGS testing. Notably, *TP53* gene mutations or deletions typically lead to aberrant expression patterns of the p53 protein. For instance, *TP53* missense mutation causes the p53 protein to escape the normal intracellular degradation process, and abnormal accumulation occurs in the nucleus of tumor cells, exhibiting diffuse expression of the p53 protein. Conversely, *TP53* termination mutation and splicing site mutation may result in loss of p53 protein expression (30). The prognostic value of p53 overexpression as a substitute for *TP53* mutation has been studied elsewhere, but the findings are inconsistent, and its clinical significance is uncertain (13, 31). However, previous studies primarily relied on first-generation sequencing methods and often did not clearly delineate different expression patterns of p53 protein or elaborate on their positive intensity. In this study, an IHC assay of the p53 protein in DLBCL was performed, and the results of *TP53* gene mutations were compared. According to its positive intensity and positive rate, it can be assigned to the negative group (<1%), low–expression group, and high–expression group (≥65%, moderate–strong staining). The p53 high–expression group and the p53 negative group were classified as MUT–p53 protein, whereas the p53 low–expression group was classified as WT–p53 protein. Preliminary results indicated that IHC can effectively predict the risk of *TP53* mutation in DLBCL. The inconsistency in some cases may be attributable to limitations of assay methods and gene post–transcriptional regulation (22, 32).

In previous studies, the value of p53 protein in clinical prognosis remains controversial (31, 33). In this study, there was no significant difference between p53–MUT (IHC) and p53–WT (IHC) in many recognized high–risk clinical stratification characteristics, including age, sex, IPI score, Han’s classification, and double expression. On the contrary, p53–MUT (IHC) was found to be more common in patients with earlier clinical stages. For the survival analysis of patients with DLBCL, although the median OS and median PFS of the mutant type were lower than those of the wild type, there was no significant difference, which is consistent with some previous findings (31). The significance of multiple TP53 mutations as a marker of poor prognosis in acute myeloid leukemia (AML) and myelodysplastic syndromes (MDS) has been widely recognized and integrated into international consensus guidelines. The concept of TP53 allelic status (mono- or biallelic mutation) has been proposed (29). However, this has not been unified for lymphoma, which is the focus of our subsequent research. This study highlights a crucial finding: DLBCL patients with p53-MUT detected via IHC exhibit challenges in achieving complete remission following standard R–CHOP treatment. Furthermore, their ORR is notably lower compared to the wild-type group. These results suggest that individuals with p53 protein mutations are at a higher risk of progressing to relapsed and refractory DLBCL. Therefore, early and timely diagnosis coupled with the adjustment of treatment strategies may lead to more favorable patient outcomes.

## Conclusions

In conclusion, this study summarizes the characteristics of *TP53* gene alterations in DLBCL and demonstrates that p53 protein (IHC) can be employed to predict *TP53* gene mutations more accurately. Hence, the interpretation method of the p53 protein in this study is deemed feasible. Furthermore, this research confirms that DLBCL patients with p53 protein mutations are prone to progress to relapsed and refractory type, so it is particularly crucial to identify this patient population in the early stage. Additionally, prognosis evaluation and personalized treatment strategies can be implemented for these patients to enhance their quality of life and prolong their survival time.

## Data Availability

The data presented in the study are deposited in the NCBI repository, accession number PRJNA1252966.

## Supplementary material

Supplementary Figure 1
**(A, B)** TP53 mutation by NGS also had no significant effect on OS and PFS. **(C–F)** There is no significant difference in OS and PFS between early-stage disease group and a late-stage disease group.
