# Supplementary figures and images for: TP53 mutation and immunohistochemical p53 expression characteristics in diffuse large B–cell lymphoma
Source: Front Oncol. 2025 Apr 28;15:1550207. doi: 10.3389/fonc.2025.1550207 (PMC12066628; doi:10.3389/fonc.2025.1550207)

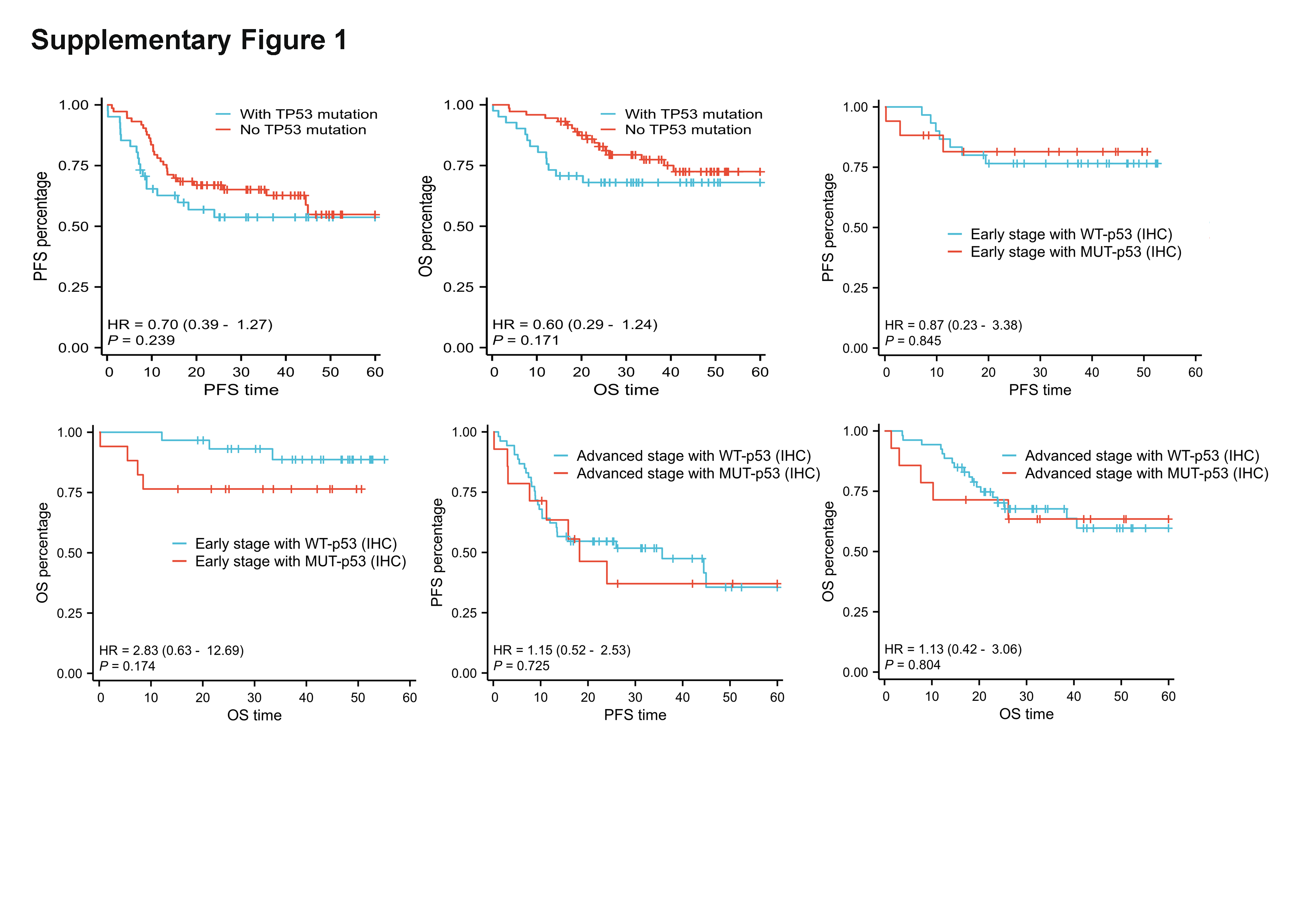

Supplement: Supplementary Figure 1 — (A, B) TP53 mutation by NGS also had no significant effect on OS and PFS. (C–F) There is no significant difference in OS and PFS between early-stage disease group and a late-stage disease group. [file Image1.tif]
