# Supplementary material for: TP53 mutation and immunohistochemical p53 expression characteristics in diffuse large B–cell lymphoma
Source: Front Oncol. 2025 Apr 28;15:1550207. doi: 10.3389/fonc.2025.1550207 (PMC12066628; doi:10.3389/fonc.2025.1550207)
Supplement: Supplementary file 2 [file Table1.docx]

**Supplementary Table 1 *TP53* Mutation type and p53 expression**

| NO. | caseAF | ExIn_ID | Function | cHGVS | pHGVS | p53 expression |
| --- | --- | --- | --- | --- | --- | --- |
| 1 | 82.77% | EX6 | missense | c.607G>C | p.V203L | High expression |
| 2 | 13.18% | EX7 | missense | c.711G>A | p.M237I | Low expression |
| 3 | 3.23% | EX8 | missense | c.814G>A | p.V272M | Low expression |
| 4 | 5.14% | EX4 | frameshift | c.304delA | p.T102Pfs*21 | Negative |
| 5 | 21.10% | EX5 | missense | c.476C>T | p.A159V | Low expression |
| 6 | 76.90% | EX5 | missense | c.422G>A | p.C141Y | High expression |
| 7 | 75.10% | EX8 | missense | c.817C>T | p.R273C | Low expression |
| 8 | 32.20% | EX7 | missense | c770T>C | p.L257P | Low expression |
| 9 | 69.77% | EX6 | nonsense | c.637C>T | p.R213* | Negative |
| 10 | 15.20% | EX7 | missense | c.711G>T | p.M237I | High expression |
| 11 | 73.71% | EX7 | missense | c.743G>A | p.R248Q | High expression |
| 12 | 42.96% | EX7 | missense | c.733G>A | p.G245S | Low expression |
| 13 | 67.04% | EX8 | missense | c.818G>A | p.R273H | High expression |
| 14 | 34.52% | EX7 | missense | c.742C>T | p.R248W | High expression |
| 15 | 55.18% | EX6 | frameshift | c.626_627delGA | p.R209Kfs*6 | Negative |
| 16 | 61.90% | EX8 | missense | c.830G>A | p.C277Y | High expression |
| 17 | 15.70% | EX4 | missense | c.358A>G | p.K120E | High expression |
| 18 | 10.50% | EX7 | missense | c.742C>T | p.R248W | High expression |
| 19 | 9.40% | EX6 | frameshift | c.630_648delCACTTTTCGACATAGTGTG | p.N210Kfs*31 | Negative |
| 20 | 33.30% | EX7 | missense | c.707A>G | p.Y236C | High expression |
| 21 | 46.40% | EX5 | frameshift | c.380_383delCCCC | p.S127Lfs*42 | Low expression |
| 22 | 6.20% | EX6 | nonsense | c.586C>T | p.R196* | Low expression |
| 23 | 31.70% | EX6 | missense | c.638G>C | p.R213P | High expression |
| 24 | 20.70% | EX7 | missense | c.743G>A | p.R248Q | Low expression |
| 25 | 51.10% | EX4 | missense | c.319T>G | p.Y107D | High expression |
| 26 | 39.90% | EX5 | missense | c.527G>A | p.C176Y | Low expression |
| 27 | 2% | EX8 | frameshift | c.861-862dupGA | p.N288Rfs*58 | Low expression |
| 28 | 12.90% | EX7 | missense | c.742C>T | p.R248W | Low expression |
| 29 | 82.10% | EX8 | missense | c.796G>A | P.G266R | High expression |
| 30 | 88% | EX5 | missense | c.157G>C | p.V173L | High expression |
| 31 | 78.40% | EX4 | missense | c.215C>G | p.P72R | Low expression |
| 32 | 63% | EX7 | missense | c.721T>C | p.S241P | High expression |
| 33 | 41.20% | EX8 | missense | c.844C>T | p.R282W | High expression |
| 34 | 75.35% | IVS9 | splice-5 | c.993+2T>G | . | Low expression |
| 35 | 75.30% | EX5 | missense | c.367G>A | p.G123S | High expression |
| 36 | 26.81% | EX6 | frameshift | c.626-627delGA | p.R209Kfs*6 | Low expression |
| 38 | 31.90% | EX8 | missense | c.817C>T | p.R273C | High expression |
| 39 | 70.10% | EX7 | missense | c.737T>C | p.M246T | High expression |
| 40 | 21.70% | EX5 | missense | c.527G>A | p.C176Y | Low expression |
| 41 | 47.40% | EX8 | missense | c.844C>T | p.R282W | High expression |
| 42 | 15.40% | EX4 | missense | c.215C>G | p.P72R | Low expression |
| 43 | 15.80% | EX5 | missense | c.389T>G | p.L130R | High expression |
| 44 | 47.50% | EX7 | missense | c.743G>A | p.R248Q | High expression |

caseAF, case allele frequency; cHGVS, complementary DNA Human Genome Variation Society; pHGVS, Protein Human Genome Variation Society.
