# Supplementary material for: TP53 mutation and immunohistochemical p53 expression characteristics in diffuse large B–cell lymphoma
Source: Front Oncol. 2025 Apr 28;15:1550207. doi: 10.3389/fonc.2025.1550207 (PMC12066628; doi:10.3389/fonc.2025.1550207)
Supplement: Supplementary file 3 [file Table2.docx]

**Supplementary Table 2 Immunohistochemical p53 expression and *TP53* mutation**

| Parameters | High p53 protein expression | Low p53 protein expression | No p53 protein expression (<1%) |
| --- | --- | --- | --- |
| *TP53* mutation | 23 | 17 | 4 |
| No *TP53* mutation | 5 | 74 | 0 |
